# DNA Repair and Ovarian Carcinogenesis: Impact on Risk, Prognosis and Therapy Outcome

**DOI:** 10.3390/cancers12071713

**Published:** 2020-06-28

**Authors:** Kristyna Tomasova, Andrea Cumova, Karolina Seborova, Josef Horak, Kamila Koucka, Ludmila Vodickova, Radka Vaclavikova, Pavel Vodicka

**Affiliations:** 1 Department of Molecular Biology of Cancer, Institute of Experimental Medicine of the Czech Academy of Sciences, Videnska 1083, 14220 Prague, Czech Republic; kristyna.tomasova@iem.cas.cz (K.T.); andrea.cumova@iem.cas.cz (A.C.); josef.horak@iem.cas.cz (J.H.); ludmila.vodickova@iem.cas.cz (L.V.); 2 Biomedical Center, Faculty of Medicine in Pilsen, Charles University, Alej Svobody 76, 32300 Pilsen, Czech Republic; 3 First Faculty of Medicine, Charles University, Institute of Biology and Medical Genetics, Albertov 4, 12800 Prague, Czech Republic; 4 Toxicogenomics Unit, National Institute of Public Health, Srobarova 48, 10042 Prague, Czech Republic; karolina.seborova@szu.cz (K.S.); kamila.koucka@szu.cz (K.K.); RVaclavikova@seznam.cz (R.V.); 5 Laboratory of Pharmacogenomics, Biomedical Center, Faculty of Medicine in Pilsen, Charles University, Alej Svobody 76, 32300 Pilsen, Czech Republic; 6 Third Faculty of Medicine, Charles University, Ruska 87, 10000 Prague, Czech Republic

**Keywords:** ovarian cancer, DNA repair, carcinogenesis, prognosis, therapy response

## Abstract

There is ample evidence for the essential involvement of DNA repair and DNA damage response in the onset of solid malignancies, including ovarian cancer. Indeed, high-penetrance germline mutations in DNA repair genes are important players in familial cancers: *BRCA1*, *BRCA2* mutations or mismatch repair, and polymerase deficiency in colorectal, breast, and ovarian cancers. Recently, some molecular hallmarks (e.g., *TP53*, *KRAS*, *BRAF*, *RAD51C/D* or *PTEN* mutations) of ovarian carcinomas were identified. The manuscript overviews the role of DNA repair machinery in ovarian cancer, its risk, prognosis, and therapy outcome. We have attempted to expose molecular hallmarks of ovarian cancer with a focus on DNA repair system and scrutinized genetic, epigenetic, functional, and protein alterations in individual DNA repair pathways (homologous recombination, non-homologous end-joining, DNA mismatch repair, base- and nucleotide-excision repair, and direct repair). We suggest that lack of knowledge particularly in non-homologous end joining repair pathway and the interplay between DNA repair pathways needs to be confronted. The most important genes of the DNA repair system are emphasized and their targeting in ovarian cancer will deserve further attention. The function of those genes, as well as the functional status of the entire DNA repair pathways, should be investigated in detail in the near future.

## 1. Introduction

Recent reports highlight the importance of DNA repair and DNA damage response (DDR), involved in the genomic instability that accompanies tumorigenesis and cancer progression [1,2,3]. Pearl et al. [4] found that every DDR process was functionally impaired to some extent in one or more cancer types. Among effector pathways of DDR, genomic alterations in DNA repair genes represent substantial changes underlying the genetics of many solid cancers e.g., breast, colorectal, and ovarian cancer (OvC) [5,6]. This paradigm is particularly pronounced in familial cancers with known germline mutations of high penetrance in DNA repair genes, e.g., breast cancer 1 and *2* (*BRCA1* and *2*) mutations in breast cancer; MutL homolog 1 (*MLH1*), MutS homolog 2 (*MSH2*), MutS homolog 6 (*MSH6*)*,* PMS1 homolog 2 (*PMS2*), and DNA polymerase epsilon (*POLE*) mutations linked to mismatch repair or polymerase deficiency in colorectal and ovarian cancers; RAD51 paralog C and paralog D (*RAD51C* and *D*) deleterious mutations and *BRCA1* mutation in OvC [6,7,8,9]. The present review article addresses the role of DNA repair machinery in OvC.

OvC is the 9th most common type of cancer and the 8th leading cause of death among female malignant diseases with an estimated annual incidence of 295,400 new cases and 184,800 deaths worldwide [10]. The majority (90%) of OvC is designated as epithelial ovarian carcinomas (EOCs) [11], divided into two major subtypes; (i) type I is composed of endometrioid, mucinous, clear cell and low grade serous ovarian carcinomas and (ii) type II includes high-grade serous ovarian carcinomas (HGSOCs) as histological dominant subtype [12]. It exhibits aggressive behavior and accounts for 70–80% of OvC deaths [13,14,15]. The other type II ovarian carcinomas present carcinosarcomas and undifferentiated carcinomas [14,16]. The present standard of care for EOC consists of optimal cytoreductive surgery and chemotherapy that includes platinum-based chemotherapy usually in combination with taxanes [17,18]. In most cases, new therapeutic approaches are tested directly against molecular targets and pathways, e.g., poly(ADP-ribose) polymerase inhibitors (PARPi) such as olaparib, rucaparib or niraparib; anti-angiogenic agents such as bevacizumab or pazopanib; inhibitors of growth factor signaling or folate pathway inhibitors; protein kinase B (AKT) signaling inhibitors; and many immunotherapeutic approaches [19,20]. Despite the advent of new treatments, long term outcomes have not significantly improved in the past 30 years with the latest five-year survival rates largely falling between 30% and 50% across the globe [21,22]. At present, the main attention is dedicated to the improvement of the overall survival (OS) of OvC patients. As stated above, the functional status of DNA repair along with DDR determines cancer onset and impacts prognosis and efficacy of chemotherapy (often acting via DNA damage generation).

## 2. Main Molecular Hallmarks of Ovarian Cancer and Association with DNA Repair System

**Figure 1:**
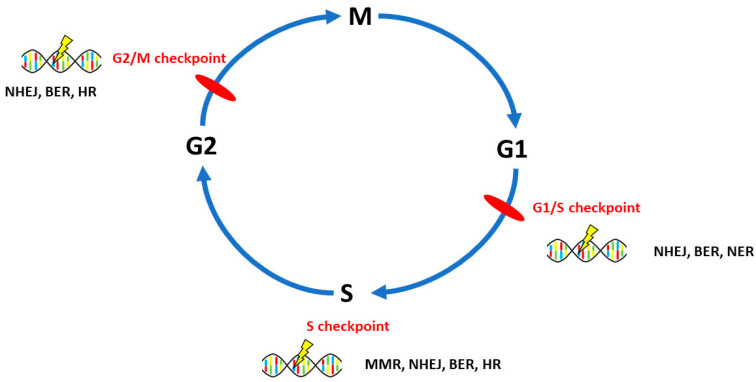
DNA repair pathways and implications in cell biology. DNA damage in the G1/S checkpoint is repaired by non-homologous end-joining repair (NHEJ), base excision repair (BER) and nucleotide excision repair (NER). In the S phase checkpoint, DNA damage is repaired by mismatch repair (MMR), homologous recombination (HR), NHEJ, BER. G2/M checkpoint DNA damage repair pathways are NHEJ, BER, HR. [29,30,31,32].

The whole system of DNA repair system is encoded by more than 150 genes and well-characterized [23]. Among existing DNA repair pathways, six pathways are implicated in OvC. In general, defective homologous recombination repair (HR), non-homologous end-joining (NHEJ), mismatch repair (MMR), base excision repair (BER), and disorders in nucleotide excision repair (NER) are typically reflected in OvC origin, pathogenesis and response to chemotherapy [20,24], whereas direct reversal of lesions is in connection with OvC addressed scarcely. Interestingly, there is sufficient evidence on the participation of all DNA repair pathways in ovarian tumorigenesis due to complex exposures from environment [25,26]. Main DNA repair pathways relevant in ovarian carcinogenesis and their role in cellular biology are illustrated in Figure 1.

In general terms of genetic profiles, tumor protein p53 (*TP53*) somatic mutations, chromosomal instability, and frequently defective HR are typical for the most usual and aggressive type II category of ovarian carcinomas largely composed of HGSOC [14]. *TP53* is a tumor suppressor which, in response to various cellular stresses (such as DNA damage, oxidative stress or hypoxia), binds to the promoter region of many genes controlling cell proliferation, apoptosis, DNA repair, etc., hereby regulates their expression [27]. 

Somatic mutations of *TP53* occur in more than half of human tumors, making it the most frequent cancer-related gene [28]. HGSOC bears *TP53* mutations in 96% of cases and about 50% of these tumors displayed defective HR due to germline and somatic *BRCA* mutations, epigenetic inactivation of *BRCA*, and abnormalities of DNA repair genes [15].

The deficiencies in MMR and *BRCA1* mutations are important hallmarks for OvC [7,8,33]. *BRCA1*/*2* germline mutations are estimated as risk factors of 10–20% of EOC [15]. Type I EOCs including low grade serous and mucinous carcinomas are typically Kirsten rat sarcoma viral oncogene homolog (*KRAS*)- and v-Raf murine sarcoma viral oncogene homolog B (*BRAF*)-mutated. Frequent mutations were also found in AT-rich interactive domain A1 (*ARID1A*), catenin beta 1 (*CTNNB1*), phosphatidylinositol-4,5-bisphosphate 3-kinase catalytic subunit α (*PIK3CA*), phosphatase and tensin homolog *(PTEN*) genes [14]. Other recently found genes in women diagnosed for EOC and associated with the risk of EOC onset are BRCA1-interacting protein C-terminal helicase (*BRIP1*), RAD50 homolog (*RAD50*), *RAD51C*, *RAD51D*, BRCA1-associated RING domain 1 (*BARD1*), checkpoint kinase 2 (*CHEK2*), meiotic recombination 11 homolog A (*MRE11A*), partner and localizer of BRCA2 (*PALB2*) and ataxia telangiectasia mutated (*ATM*) gene (as summarized in [20]).

Particularly, deleterious mutations in *RAD51C* and *RAD51D* (genes involved in HR) have been shown to confer the risk of EOC implicating their use alongside *BRCA1* and *BRCA2* in routine clinical genetic testing [9]. Except for the association of DNA repair genes variations with modulating EOC risk, some recent studies overviewed the involvement of DNA damage repair pathways in EOC progression and therapeutic response. For instance, deficiency in HR, often occurring in OvC, was associated with worse outcomes in other solid cancers [34]. Nevertheless, except for the U.S. Food and Drug Administration (FDA) agency-approved treatment of germline *BRCA*-mutated OvC or maintenance treatment of platinum-sensitive relapsed *BRCA*-mutated EOC patients by PARPi [35], other DNA repair genes and pathways are not used as therapeutic targets in clinical practice at present. Recent period witnessed approaches with utilization of different kinds of DNA damage (repaired by different DNA repair pathways) induced simultaneously in frame of combinational chemotherapy (e.g., radiation and chemotherapy, the use of natural compounds in parallel with cytostatics [36]). These concepts are believed to diminish adverse effects of chemotherapeutics and postpone the advent of resistance. The role of genes and pathways of DNA repair system in ovarian carcinogenesis, prognosis, therapy response, and their potential as possible therapeutic targets are the main focuses of this review.

## 3. DNA Repair Pathways Involved in the Onset, Progression and Prognosis of Ovarian Cancer

### 3.1. Homologous Recombination Repair

**Figure 2:**
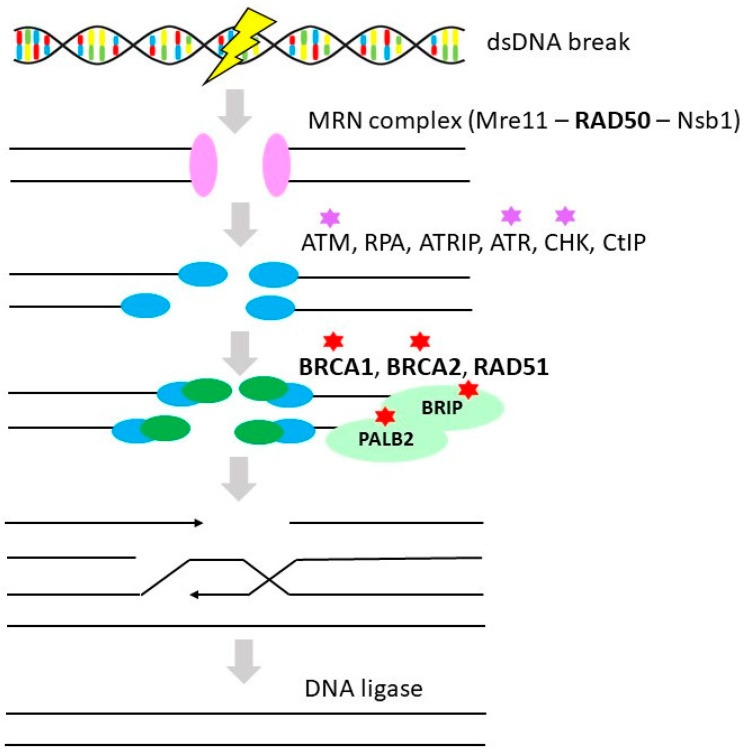
Homologous recombination. Simplified scheme of homologous recombination in double-strand DNA breaks and DNA inter-strand crosslinks (gene alternations in OvC in **bold**, therapeutic interventions considered in OvC therapy marked by a red star (PARP inhibitors), purple star (check-point inhibitors)) [43]. Protein names: meiotic recombination 11 (MRE11), RAD50 homolog (RAD50), Nijmegen breakage syndrome 1 (NBS1), Ataxia telangiectasia mutated (ATM), replication protein A (RPA), Ataxia telangiectasia and RAD3 related-interacting protein (ATRIP), Ataxia telangiectasia and RAD3 related (ATR), checkpoint kinases (CHEK), retinoblastoma binding protein 8 (CtIP), breast cancer 1 and 2 (BRCA1 and 2), RAD51 homolog 1 (RAD51), BRCA1-interacting protein C-terminal helicase (BRIP1), partner and localizer of BRCA2 (PALB2) [44].

HR is an essential high-fidelity DNA repair pathway, which provides template-dependent repair of complex DNA damage including DNA gaps, DNA double-strand breaks (DSBs), and DNA inter-strand crosslinks (repair mechanism is illustrated in Figure 2). It has also a prominent role in DNA replication and telomere maintenance. HR is active during S and G2 phases of the cell cycle when the sister chromatid is available and serves as a template. Normal cellular processes during DNA replication (due to replication fork collapse or arrest) and meiosis (during the process of crossing-over) may also produce DNA damage, taken care of HR. However, a variety of exogenous agents can induce DNA damage employing HR such as radiation, UV light, and crosslinking agents (e.g., platinum derivatives) [37,38].

Unrepaired DSBs are considered to be the most deleterious and fatal for DNA integrity. Defects in HR may result in deletions, translocations, duplications, loss of heterozygosity or aneuploidy [39]. Consequently, defective HR is linked to various types of cancers, especially OvC and breast cancer.

The defective HR pathway is found in about 50% of HGSOCs. However, non-serous histological types including clear cell, endometrioid, and carcinosarcomas have also been shown to harbor alterations in HR [40]. In OvC, HR deregulation is driven mostly by somatic and germline mutations in high-penetrance susceptibility genes *BRCA1/2* [41,42].

BRCA1 and BRCA2 proteins play crucial roles in repairing DBSs. The deficiency of BRCA1 or 2 is caused by germline or somatic loss of function mutations (mainly deletions) in *BRCA1/2* genes or by hypermethylation of the *BRCA1* promoter. BRCA1 is active in the early phases of HR and binding sites for multiple proteins acts as a scaffold that organizes other repair proteins to the site of the repair. BRCA2 acts later and is responsible for the loading of RAD51 onto replication protein A (RPA)-coated DNA.

Mutations in *BRCA1* and *BRCA2* genes are associated with a high risk of hereditary breast cancer and OvC. From a current prospective study of 9856 *BRCA* mutation carriers, the cumulative risk for OvC to age 80 was 44% for *BRCA1* mutation carriers and 17% for *BRCA2* mutation carriers [45]. *BRCA1* mutation carriers develop OvC earlier compared to *BRCA2* mutation carriers (mean age at the diagnosis for *BRCA1*-mutation carriers is 51.3y, for *BRCA2*-mutation carriers 61.4y) [46], while typical age at the diagnosis for the general population is about 63 years [47,48]. The risk for OvC varies also with the type and the location of *BRCA* gene mutations. Results suggest that there are “ovarian cancer cluster regions” (OCCRs) that lie in or near exons 11 of both genes and mutations in these regions are associated with OvC rather than with breast cancer [49]. Additionally, “breast cancer cluster regions” (BCCRs) were identified in both genes as well, predisposing mainly to breast cancer and suggesting different mutation spectrum for ovarian and breast cancer [49].

Pooled analysis of several OvC studies revealed that *BRCA1/2* mutation carriers exhibit significantly improved survival compared to non-carriers. This effect is pronounced in *BRCA2*-mutation carriers. The five-year survival rate in non-carriers was 36%, 44% for *BRCA1*-mutation carriers, and 52% for *BRCA2*-mutation carriers [50]. The survival advantage may be partly related to their enhanced sensitivity to platinum-based chemotherapy, which is conventionally used as a first-line OvC chemotherapy. Interestingly, epigenetic silencing of *BRCA1* through promoter hypermethylation was not associated with better response to platinum-based chemotherapy and with improved survival in HGSOC patients [51]. However, *BRCA1/2*-mutated tumors are more likely to develop distant metastases. This may be partly related to the high degree of genomic instability present in these tumors [52].

Patients with HR-deficient OvC exhibit significantly higher response rates and prolonged progression-free survival (PFS) following platinum-based chemotherapy [50,53]. Even after disease recurrence, HR-deficient OvCs exhibit good response for other lines of platinum chemotherapy, while other OvCs often acquire chemo-resistance [54]. Nowadays, several PARPi are used in the treatment of *BRCA*-mutated OvC. Their cytotoxic effect is based on the synthetic lethality principle, where PARPi kill cancer cells with defective HR. The response to olaparib, the first FDA-approved PARPi, is the best in germline *BRCA*-mutated platinum-sensitive OvC and the worst in wild-type (wt) *BRCA* platinum-resistant OvC [55]. However, patients with *BRCA*-mutated OvC may develop resistance towards PARPi through multiple mechanisms including somatic reversion mutations of *BRCA* genes, reversion of *BRCA*-promoter methylation, overexpression of hypomorphic *BRCA*, decreased poly(ADP-ribose) polymerase 1 (*PARP1*) expression due to de novo mutations, drug efflux, acquisition of new mutations in/silencing of other DNA repair genes. These mechanisms lead to either restoration of HR or protection of replication fork [56].

Other HR pathway alterations include medium penetrance mutations in several Fanconi anemia genes (mainly *PALB2* and Fanconi anemia complementation group A, C, I, and L (*FANCA*, -*C*, *-I*, and -*L*), in RAD genes (such as *RAD50*, RAD51 homolog 1 (*RAD51*), *RAD51C, RAD51D* and RAD54-like (*RAD54L*)), and in DDR genes involved in HR (*ATM*, Ataxia telangiectasia and RAD3 related (*ATR*), checkpoint kinase 1 (*CHEK1*), and *CHEK2*) [43].

In particular, mutations in *RAD51C* and *RAD51D* have been associated with the risk of EOC, having potential use in routine clinical genetic testing [9]. *RAD51* homolog genes are considered to be moderate penetrance OvC susceptibility genes, responsible for about 1% of OvC cases. Both proteins are important parts of the complex named BCDX2 (together with RAD51 paralog B (RAD51B) and X-ray repair cross-complementing 2 (XRCC2)) which is required for the formation of RAD51 foci in response to DNA damage. Biallelic mutations in *RAD51C* gene are present in Fanconi anemia-like syndrome [57]. Mutations in *RAD51* genes are usually of deleterious type or hypermethylation of the *RAD51C* promoter [58].

Various studies disclosed strikingly elevated risk for OvC, reflected by odds ratio for *RAD51C* mutations ranging from 5 to 12 [59,60]. Similar odds ratios (5 to 12) have been assessed for mutations in *RAD51D* [9,61,62,63]. The lifetime risk for developing OvC for *RAD51D* mutation carriers is estimated to be 10–15% [62].

In the recent study, the median age at diagnosis in *RAD51C* and *RAD51D* mutation carriers was 39 and 32.5 years respectively, suggesting the involvement of *RAD51* genes mutations in earlier onset of OvC [60].

Available results demonstrate that *RAD51C* and *RAD51D* are OvC predisposition genes, but further studies should evaluate their exact contribution to the OvC risk and onset.

Current studies suggest that mutations in *RAD51* paralogs predispose ovarian tumors to be sensitive to PARPi. In vivo study on the patient-derived xenograft mice model revealed that *RAD51C* promoter methylation predisposes to the sensitivity of ovarian tumors to niraparib (PARPi) [64]. Primary mutations in *RAD51C* and *RAD51D* confer to PARPi rucaparib sensitivity and, on the other hand, reverse secondary mutations in these genes contribute to acquired PARPi resistance [65].

RAD50 is a part of the so-called MRN complex (consisting of meiotic recombination 11 (MRE11), RAD50, and Nijmegen breakage syndrome 1 (NBS1)) which is essential for response to DSB damage and HR initiation. Heeke et al. identified mutations in HR genes in several types of solid tumors including OvC and found that *RAD50* is mutated in about 0.12% of tumors [66]. Interestingly, immunohistochemical detection of MRN complex revealed that 41% of epithelial low-grade OvC lacked MRN complex and 10.3% of tumors lacked RAD50 specifically. The role of *RAD50* mutation on OvC risk and onset must, therefore, be further evaluated [67].

Kessous et al. correlated the survival of OvC patients with expression profiles of different HR genes and found that expression of *RAD50* correlates with better PFS [68]. In *BRCA*-wt OvC patients, 18% of patients exhibit *RAD50* copy number deletion which was associated with significantly better OS and PFS [69].

According to an in vitro study from Zhang et al. [69], knockdown of *RAD50* gene expression in OvC cell lines was associated with better response to PARPi (olaparib and rucaparib). Further research may help to better define the group of patients who may profit from PARPi, even if they are *BRCA*-wt but simultaneously have deficient other steps of HR pathway.

PALB2 is another important member of HR, interacting with BRCA2 as well as with BRCA1 and several members of the DDR family [70]. Its mutations are associated with an elevated risk of developing several cancers including breast cancer [71]. A polish study on 460 *BRCA*-wt OvC patients revealed that 1.5% of patients had germline deletion in the *PALB2* gene [72]. A recent study on 524 families from 21 countries harboring pathogenic variants of the *PALB2* gene estimated the relative risk of OvC to be nearly 3. The estimated risk of developing OvC to age 80 is almost 5% [73].

Studies of therapy outcome suggest that *PALB2*-deficient ovarian tumors, similarly to other HR deficient OvC, may benefit from PARPi therapy [74]. In vivo study on pediatric cancers suggests that *PALB2* mutations are associated with exceptional response to talazoparib in mouse xenografts [75].

BRIP1 is another member of HR pathway with ATPase and helicase activity known for its role in OvC predisposition. It was previously associated with breast cancer risk [76,77,78,79] however, results from these studies are inconsistent and several other studies found no association of *BRIP1* mutations and breast cancer risk [80,81]. It is one of the most common OvC susceptibility genes with 0.9–2.5% frequency in all patients carrying a mutation in this gene [62,82,83,84]. A study from Weber-Lassalle et al. on the loss of function *BRIP1* mutations found that these mutations confer a high OvC risk in familial OvC patients as well as in late-onset OvC patients (OR = 20.97 and 29.91 respectively) [83]. Another study assessed the relative risk (RR) of EOC being 11.22 (95% confidence interval [CI] = 3.22 to 34.10, *P* = 1 × 10^−4^) and cumulative risk of developing EOC by age 80 years to 5.8% (95% CI = 3.6% to 9.1%) making it a moderate risk factor for OvC [82].

Similarly, as in other members of HR pathway, mutations in *BRIP1* are believed to predispose OvC tumors to better respond to both PARPi and platinum [55].

The overall DNA repair system is tightly coordinated with cell cycle checkpoints as an essential part of DDR. A large recent genome-wide association study (GWAS) identified an association of *CHEK2* gene variants with EOC risk. CHEK2 is a serine-threonine kinase which, in response to DSB, phosphorylates serine 988 in BRCA1 [85]. This phosphorylation is required for the formation of BRCA1–PALB2–BRCA2 effector complex critical in RAD51-mediated HR [86,87]. According to the GWAS, the strongest association showed *CHEK2* single-nucleotide polymorphism (SNP) rs17507066 with serous EOC. The authors reported an additional association of *CHEK2* rs6005807 with HGSOC. Both SNPs, i.e., rs17507066 and rs6005807 showed linkage disequilibrium r^2^ = 0.84 [88]. Additionally, *CHEK2* gene variant rs6005807 was associated with EOC risk (irrespective of *BRCA1/BRCA2* mutations) in an independent, large GWAS study of Phelan et al. (for detailed description of SNPs discussed in our review see Table 1) [89].

### 3.2. Non-Homologous End-Joining

**Figure 3:**
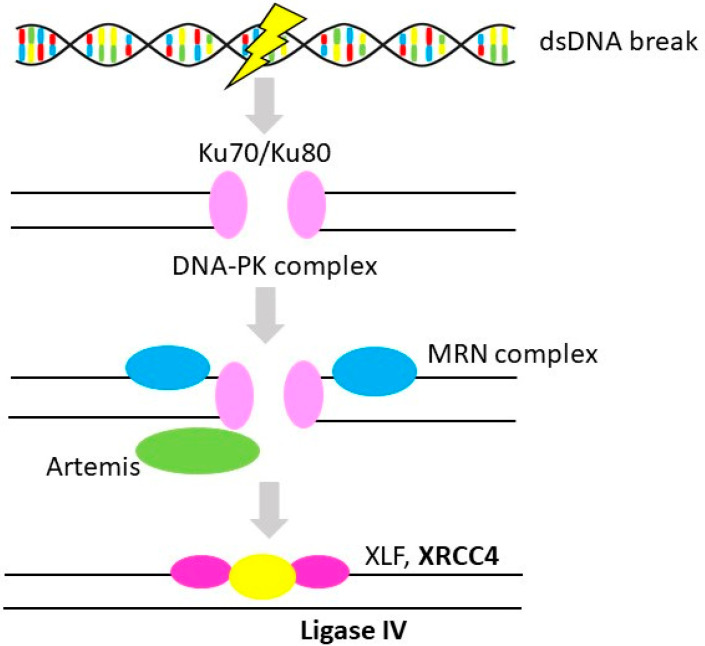
Non-homologous end-joining repair. Simplified scheme of non-homologous end-joining repair of double strand DNA breaks (gene alterations in OvC in **bold**) [32]. Protein names: DNA end-binding proteins Ku70/Ku80 (Ku70/Ku80), DNA-dependent protein kinase (DNA-PK), MRE11-RAD50-NBS1 complex (MRN complex), artemis (DCLRE1C), X-ray repair cross complementing-like factor. (XLF), X-ray repair cross complementing 4 (XRCC4), DNA ligase 4 (LIG4) [44].

NHEJ is a most robust pathway which repairs DSBs in DNA. Unlike HR, DNA lesions are directly ligated without a need of a homologous template. Since it doesn’t require sister chromatid (available during S and G2 phases), it may be executed throughout the entire cell cycle (repair mechanism is illustrated in Figure 3). In fact, NHEJ appears to repair almost all DBSs outside the S and G2 phases of the cell cycle and about 80% during the S and G2 phases [97]. However, since the process involves losses of sequences during the junction formation, NHEJ is a potentially a mutagenic process. Apart from its key role in repairing DSBs, NHEJ is an essential part of adaptive immunity during V(D)J recombination, giving rise to a highly diverse repertoire of immunoglobulins and T cell receptors.

Germline mutations in genes involved in NHEJ are associated with severe immunodeficiency and developmental abnormalities [98,99], genomic instability as well as with different cancers, such as leukemias or bladder cancer [100,101,102,103]. Whereas excessive research has been done on HR and OvC, less is known about the relationship between mutations in NHEJ genes and OvC. McCormick et al. assessed NHEJ in a panel of OvC cell lines and 47 primary OvC cell cultures. This study shows that about 40% of OvC cell lines and primary cultures were defective in NHEJ, independently of HR [104]. Interestingly, NHEJ-deficient cell lines and cell cultures were resistant to rucaparib (PARPi). Sensitivity to this PARPi was observed only in NHEJ-competent/HR-deficient cultures, potentially explaining why some HR-deficient tumors are resistant to PARPi.

Probably because of its predominant role in DSBs repair, mutations in NHEJ genes are less common and only a minor part of human cancers are associated with their loss or alterations. X-ray repair cross-complementing 4 (XRCC4) and DNA ligase 4 (LIG4) were two members of the NHEJ pathway studied in association with OvC.

XRCC4 protein is involved in the ligation phase of NHEJ pathway. Mutations in *XRCC4* have been linked mainly to developmental disorders as microcephaly and dwarfism [105]. However, high expression of *XRCC4* has been linked also to the poor outcome of OvC patients, making it one of the candidate biomarkers for OvC [106]. 

LIG4 is an essential protein in NHEJ, making a complex with XRCC4. Mutations in *LIG4* are a cause of rare autosomal recessive LIG4 syndrome. Polymorphisms in the *LIG4* gene have been associated with increased risk for several cancers [107]. Currently, there is insufficient evidence that *LIG4* gene variants are involved in OvC risk or prognosis. A SNP rs1805386 in *LIG4* was believed to be associated with OvC risk, but this association was later dismissed [108]. 

### 3.3. Mismatch Repair

**Figure 4:**
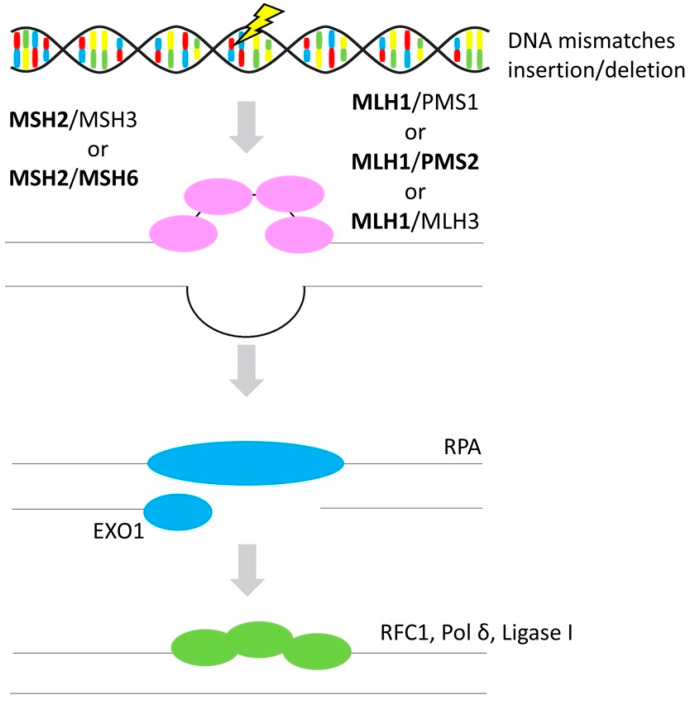
Mismatch repair. Simplified scheme of mismatch repair of DNA mismatches or insertion/deletions mispairs (gene alternations in OvC in **bold**) [111]. Protein names: MutS homolog 2 (MSH2), MutS homolog 3 (MSH3), MutS homolog 6 (MSH6), MutL homolog 1 (MLH1), PMS1 homolog 1 (PMS1), PMS1 homolog 2 (PMS2), MutL homolog 3 (MLH3), replication protein A (RPA), exonuclease 1 (EXO1), replication factor C subunit 1 (RFC1), DNA polymerase delta (POLD), DNA ligase 1 (LIG1) [44].

Besides the *BRCA1* and *BRCA2* mutations, MMR deficiency is the most common cause of hereditary OvC [109]. MMR system corrects DNA base mismatches in newly replicated DNA which were not recognized by DNA replication machine, or insertion/deletion mispairs as is illustrated in Figure 4. MMR acts mainly in the S phase of the cell cycle [110].

In humans, seven genes are implicated in the MMR system. Mismatch recognition is mediated by a heterodimer, composed of MSH2 and MutS homolog 3 (MSH3), or MSH2 and MSH6. This heterodimer then interacts with another heterodimer, composed of MutL homologs MLH1 and PMS1 homolog 1 (PMS1), MLH1 and PMS2, or MLH1 and MutL homolog 3 (MLH3), which forms single-stranded nicks on either side of the mismatch [112].

Germline mutations in MMR genes *MLH1*, *MSH2*, *MSH6*, and *PMS2* or loss of expression of *MSH2* cause Lynch syndrome, also known as hereditary non-polyposis colorectal cancer [113]. Depending on the particular MMR gene, this multi-cancer syndrome increases the cumulative lifetime risk of OvC from 6% to 12% [114].

Characteristic molecular signature occurring as a result of inactivation of the DNA MMR is called microsatellite instability (MSI) [115]. It is a hypermutable phenotype manifested through alterations in the size of repetitive DNA sequences. Tumor profiling for MSI serves as a measure for the personalized management of several cancers [116,117]. Regarding OvC, MSI occurs in a limited percentage of the tumors (2–20%) and affects predominantly endometrioid (19.2%), mucinous (16.9%), clear cell (11.2%), and serous (7.9%) subtypes [118,119]. Both endometrioid and clear cell subtypes with MSI show increased levels of tumor-infiltrating lymphocytes and thus may be susceptible to immune checkpoint inhibitor monotherapy [120]. In a very recent study of 478 OvCs by Fraune et al., MMR deficiency occurred almost exclusively in endometrioid subtype (8 of 32) and also in one of 358 serous carcinomas. MMR of other subtypes (mucinous, clear cell, carcinosarcomas of Mullerian origin, and mixed carcinosarcomas) was functional [121]. Whereas all MMR-deficient endometrioid cancers were MSI and showed loss of MLH1/PMS2 proteins in five of 32 cases, MSH2/MSH6 in two of 32 cases, and isolated MSH6 in one of 32 cases; the MMR-deficient serous carcinoma was microsatellite stable and showed PMS2 protein loss and an altered pattern of MLH1 with putative partial MLH1 protein loss [121].

Earlier studies suggested the role of MMR in signaling that triggers apoptotic activity [122]. This was further confirmed by the proof that MMR-deficient cells can continue to proliferate despite DNA damage [123]. The chemical nature of platinum derivatives may explain the resistance as well. They induce, by attacking -SH, -NH and -OH nucleophilic centers of DNA bases, either monofunctional N7-guanine adducts (minor product) or the bifunctional adducts resulting in guanine-guanine intrastrand crosslinks, guanine-adenine intrastrand crosslinks (both representing a majority of lesions) and guanine-guanine interstrand crosslinks of two nonadjacent guanines. Arising crosslinks have inhibitory effects on transcription and replication. Platinum derivatives may also bind to nucleophilic centers of proteins, forming various crosslinks that affect further their function [2,124].

Epigenetic events underlying MMR deficiency have also been investigated. As for sporadic cancers (OvC included), compromised MMR function due to promoter hypermethylation is known in *MLH1* and *MSH2*. Resistance to platinum in EOC has been associated with hypermethylation of the *MSH2* upstream region [125]. Thus, lower expression of *MSH2* may indicate the poor prognosis in EOC patients [125]. In secondary EOC, *MLH1* hypermethylation was found to be a cause of acquired platinum resistance as well, and it occurred more frequently in tumors treated with four or more courses of platinum-based chemotherapy [126]. The exact role of *MLH1* and *MSH2* in the platinum resistance is not yet clear. Watanabe et al. suggest that methylation of *MLH1* during the platinum chemotherapy may be a temporary change protecting cancer cells from cytotoxic agent-induced apoptosis because after a platinum-free interval of 6- to 12- months, they become sensitive to platinum agents again [126]. Moreover, Zhao et al. reported that a sufficient MMR system, defined in their study by high mRNA levels of *MSH6*, *MLH1*, and *PMS2*, may indicate better OS in OvC treated with platinum-based chemotherapy [127].

### 3.4. Base Excision Repair

**Figure 5:**
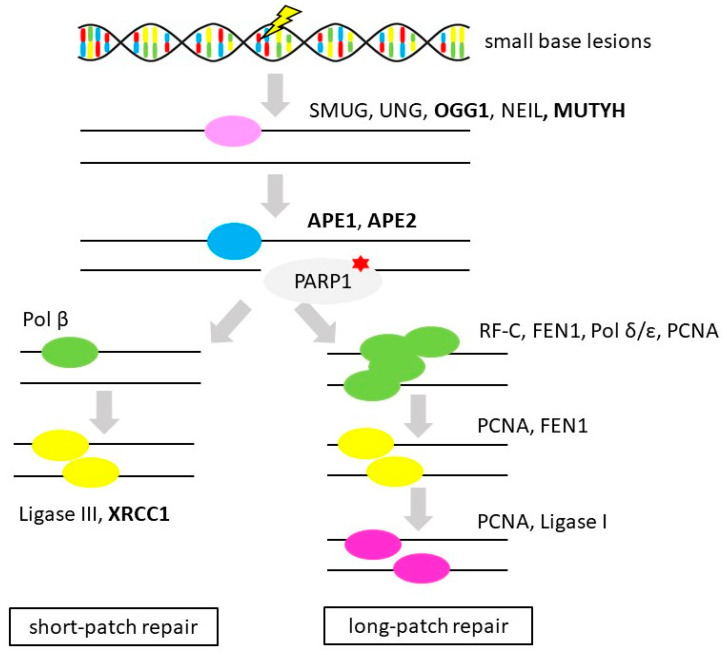
Base excision repair. Simplified scheme of base excision repair of small base lesions (gene alternations in OvC in bold, therapeutic interventions considered in OvC therapy marked by red star (PARP inhibitors)) [128]. Protein names: single-strand selective monofunctional uracil DNA glycosylase (SMUG), uracil DNA glycosylase (UNG), 8-oxoguanine DNA glycosylase 1 (*OGG1*), endonuclease VIII-like (NEIL), MutY DNA glycosylase (MUTYH), apurinic/apyrimidinic endonuclease 1 and 2 (APE1 and 2), poly(ADP-ribose) polymerase 1 (PARP1), DNA polymerase beta, delta and epsilon (POLB, -D, -E), DNA ligase 1 and 3 (LIG1 and 3), X-ray repair cross-complementing 1 (XRCC1), replication factor C (RFC), Flap endonuclease 1 (FEN1), proliferating cell nuclear antigen (PCNA) [44].

BER is an essential part of DNA repair machinery, which is responsible for repairing small base lesions (alkylations, oxidations, deaminations, depurinations or single-strand breaks (SSBs)) resulting from endogenous (products of metabolism) as well as exogenous (radiation, chemicals, drugs) sources of damage. BER consists of several components; DNA glycosylases, apurinic endonucleases (such as apurinic/apyrimidinic endonuclease 1 (APE1)), DNA polymerases (such as DNA polymerase beta (POLB)), Flap endonuclease 1 (FEN1) and DNA ligase (DNA ligase 1 or 3 (LIG1 or 3)). Other important players participating in BER are PARP1 or X-ray repair cross-complementing 1 (XRCC1) (as is illustrated in Figure 5).

DNA glycosylases (e.g., single-strand selective monofunctional uracil DNA glycosylase (SMUG), uracil DNA glycosylase (UNG), 8-oxoguanine DNA glycosylase 1 (OGG1) or endonuclease VIII-like (NEIL) DNA glycosylases) initiate the BER pathway. Depending on the type of lesion, one of the 11 glycosylases is used to excise the affected base. Impairment of these glycosylases is often linked with various cancers, such as colorectal, oesophageal, gastric, ovarian or lung cancer [129,130]. In the term of OvC, SNPs in the *OGG1* gene were described to increase the risk of OvC (for more details see Table 1). OGG1 is responsible for the excision of 8-oxoguanine, which is the result of damage caused by reactive oxygen species. Polymorphism Ser326Cys (rs1052133) was identified as a risk factor in 720 OvC patients compared to 720 healthy controls from Poland [90] and in a Chinese population using 420 patients and 840 controls, where they also linked it with type II EOC [91]. It is also known that Ser326Cys is linked with decreased repair capacity to oxidative damage [131]. Another polymorphism in *OGG1* (rs2304277) increased the risk of OvC in *BRCA1* mutations carriers [92]. In the following work they described rs2304277 role in *OGG1* downregulation and a possible contribution to telomere shortening [132]. Other studies support that *OGG1* downregulation leads to telomere shortening [133]. MutY DNA glycosylase (MUTYH) excises adenine, which is inappropriately paired. It is known especially for its role in an increased risk of colorectal cancer [134], but its biallelic mutation is also a risk factor for OvC [135]. The role of DNA glycosylases in the therapy outcome of OvC remains unknown.

APE1 and apurinic/apyrimidinic endonuclease 2 (APE2) cleave the apurinic/apyrimidinic sites left by the glycosylases or by spontaneous depurination [136,137]. APE1 is the major apurinic endonuclease in humans with more than 95% total cellular activity leaving the rest for APE2 [138]. There was identified SNP (rs1130409), which was significantly associated with risk for OvC [93]. The higher level of APE1 was reported in serous and mucinous tumors. Moreover, APE1-positive cases had a lower chance of ideal debulking surgery with consequent worse OS, implicating a more aggressive phenotype [139]. Cellular localization of APE1 had its impact on disease prognosis as well. Cytoplasmatic localization was higher in EOCs stages III and IV in comparison with lower stages in FIGO classification and it was, also, linked with lower survival rate [140]. Moreover, the abnormal cytoplasmatic level of APE1 with an abnormal level of nucleophosmin (NPM1) is associated with poor prognosis and higher chemoresistance of HGSOC [141].

XRCC1 is a scaffolding protein, which interacts with PARP1 and LIG3 in BER pathway. It has no enzymatic activity but acts as a scaffold allowing other repair proteins to carry out their enzymatic work [138]. Several studies studied OvC risk and polymorphisms in the *XRCC1* gene. Polymorphism Arg399Gln (rs25487) is linked with higher susceptibility to OvC development [94]. Association between the same polymorphism [95] along with Arg194Trp (rs1799782) [95,96] and worse clinical outcome and prognosis was also found. The expression of *XRCC1* was significantly linked with a higher stage of the illness, serous histological type of tumor, sub-optimal debulking surgery, and platinum resistance. All of these lead to a higher risk of death and worse prognosis [142].

PARP1 has become one of the major topics in BER in the last decade. Its role in BER is the detection of single-strand breaks and PARP1 acts as a signal for the repair machinery, which consists of scaffolding protein XRCC1, LIG3, and POLB. In 2014, PARPi were approved by FDA and European Medicine Agency (EMA) for use in OvC. PARPi effect is mediated by its synthetic lethality concept in HR deficient cells. Inhibition of PARP1 promotes SSBs, which, if unrepaired, consequently lead to DSBs. HR deficiency causes reliance on error-prone NHEJ pathway, therefore with PARPi together lead to genetic damage followed by cell death [143]. As for OvC, present approved application of PARPi is for patients with germline *BRCA1/2* mutations, for patients with germline or somatic mutation *BRCA1/2* with relapsed illness or patients with relapsed illness sensitive to platin-derivate chemotherapy regardless to *BRCA* status. Other indications are under clinical trials and have not been approved yet. There is a growing number of studies pointing out the potential benefit of PARPi treatment in other DDR genes deficiency outside *BRCA* mutations (e.g., *ATM*, *ATR*, *BARD1*, *BRIP1*, *CHK1*, *CHK2*, *PALB2*, *RAD51* or *FANC*) or combination treatment with other chemotherapeutics and targeted therapy. For more information, the reader is referred to other excellent up to date reviews focused on *PARP* and its inhibitors [144,145,146,147,148]. However, in vitro and in vivo evidence suggest that mutations in *PARP1* abolishing the DNA binding cause the resistance towards PARPi [149].

### 3.5. Nucleotide Excision Repair

**Figure 6:**
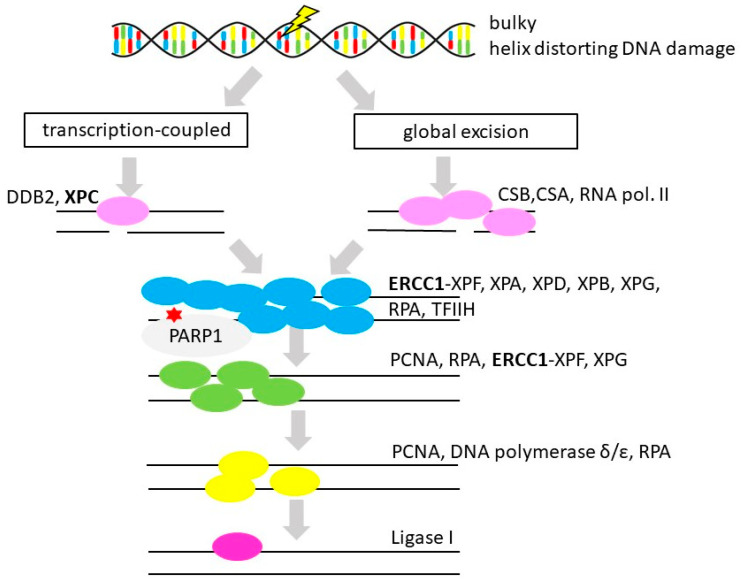
Nucleotide excision repair. Simplified scheme of nucleotide excision repair of bulky lesions and helix distorting DNA damage DNA (gene alternations in OvC in **bold,** therapeutic interventions considered in OvC therapy marked by a red star (PARP inhibitors)) [150]. Protein names: damage specific DNA binding protein 2 (DDB2), xeroderma pigmentosum complementation group A, B, C, D, F, G (XPA, -B/ERCC3, -C, -D/ERCC2, -F/ERCC4, -G), Cockayne syndrome A and B (CSA and B), RNA polymerase II (RNA pol. II), excision repair cross-complementation group 1 (ERCC1), replication protein A (RPA), transcription factor II Human (TFIIH), poly(ADP-ribose) polymerase 1 (PARP1), proliferating cell nuclear antigen (PCNA), DNA polymerase delta and epsilon (POLD and E), DNA ligase 1 (LIG1) [44].

NER recognizes bulky, helix distorting DNA damage, the main of which include UV photoproducts, polycyclic aromatic hydrocarbons, aromatic amines, platinated products, and several others (repair mechanism is illustrated in Figure 6). The deficiency of several proteins in the NER pathway is tied to three rare autosomal recessive syndromes: Cockayne syndrome, Xeroderma pigmentosum and the photosensitive form of the trichothiodystrophy [150]. Cockayne syndrome is a neurodegenerative disease caused by the mutation in either Cockayne syndrome A (*CSA*/*ERCC8*) or Cockayne syndrome B (*CSB*/*ERCC6*) genes, which leads to impaired transcription-coupled NER [151]. Xeroderma pigmentosum, characterized by extreme photosensitivity to UV radiation, results from the mutations in any of genes xeroderma pigmentosum complementation group A, B, C, D, E, F, G (*XPA*, -*B*/*ERCC3*, -*C*, -*D*/*ERCC2*, -*E*, -*F*/*ERCC4*, -*G*), xeroderma pigmentosum variant (*XPV*), or excision repair cross-complementation group 1 (*ERCC1*) [152]. Trichothiodystrophy belongs to ectodermal disorders. About half of the patients are photosensitive because they bear the mutation in *XPB*/*ERCC3*, *XPD*/*ERCC2*, or general transcription factor IIH subunit 5 (*GTF2H5*) [153].

Zhao et al. analysed 17 SNPs in NER genes *XPA, XPC, XPD*/*ERCC2*, *XPF*/*ERCC4, XPG*, and *ERCC1* in 89 OvC cases and 356 controls, and their results suggested that *ERCC1, XPC*, and *XPD*/*ERCC2* may be linked to OvC susceptibility [154]. Although this study for the first time explored the association of core genes in NER pathway with OvC, it should be pointed out that the sample size was insufficient to link OvC susceptibility to particular genetic variations and further, authors were not able to measure the mRNA expression of *ERCC1, XPC*, and *XPD*/*ERCC2* to validate their findings. However, the study by Sun et al. associated higher *XPC* mRNA expression with poor OvC prognosis [155]. ERCC1 is a non-catalytic subunit of 5′ endonuclease which in complex with XPF/ERCC4 (a catalytic subunit) incises the damaged DNA strand on the 5′ side of the lesion. XPC initiates NER reaction by detecting the DNA damage. XPD/ERCC2 is a helicase that, as a part of TFIIH core complex, unwinds (together with other TFIIH helicase XPB/ERCC3) DNA around the site of the lesion to enable its subsequent incision.

Cisplatin regimen is a standard chemotherapeutic procedure for OvC patients [156]. The most prominent damage the cisplatin introduces in DNA are 1,2- and 1,3-intrastrand crosslinks, which can be removed by NER. It is hardly surprising, therefore, that upregulation of NER mediates resistance to cisplatin-based therapy [2,157]. Ishibashi et al. reported that in OvC cell lines, tyrosine kinase with immunoglobulin-like and EGF like domains 1 (TIE1) promotes XPC-dependent NER and this leads to decreased susceptibility to cisplatin-induced cell death [158]. Last but not least, a study in 559 EOC patients showed an association of *ERCC1* polymorphisms rs11615 and rs3212986 with cisplatin resistance [159]. Beyond the PARP1 well-established role in BER, this protein is also known to regulate the NER system by its association with XPA (for more details, see Section 4) [160].

### 3.6. Direct Repair

**Figure 7:**
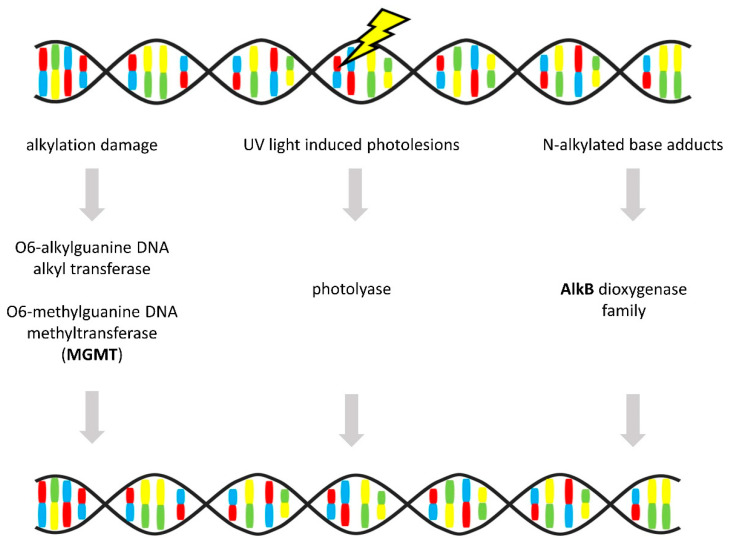
Direct repair. Simplified scheme of direct lesion reversal removing alkylation and UV-induced damage, and N-alkylated base adducts (gene alternations in OvC in **bold**) [162]. Protein names: O^6^-methylguanine DNA methyltransferase (MGMT), alpha-ketoglutarate-dependent dioxygenase AlkB (AlkB) [44].

Unlike other DNA repair mechanisms, a direct reversal of a lesion represents a relatively simple way to remove some DNA and RNA modifications, e.g., at guanine O^6^ position, without incision of phosphodiester backbone, DNA synthesis, and ligation. The base damage is eliminated in single enzyme reactions, allowing error-free repair (illustrated in Figure 7). The most common modifications involve DNA alkylation damage or RNA methylation arising by epigenetic mechanisms [44,161].

As an alternative to complex NER mechanism works photoreactivation [163]. This direct repair mechanism is mediated by photolyases and removes ultraviolet light-induced modifications of DNA, namely cyclobutane pyrimidine dimers and pyrimidine-pyrimidone photoproducts. Placental mammals possess however no class of photolyases and are reliant only on NER [164].

In humans, enzymes directly employed in DNA repair of alkylation damage are O^6^-methylguanine DNA methyltransferase (MGMT), which erases alkylations at the O^6^ position of guanine and thus prevents DNA cross-links, and alpha-ketoglutarate-dependent dioxygenase AlkB (AlkB) homologs, which oxidatively dealkylate e.g., N^1^-methyladenine, N^6^-methyladenine or N^3^-methylcytosine [161,165].

Downregulation of *MGMT* expression due to hypermethylation of its CpG islands located in the promoter region of *MGMT* and its probable relation to OvC carcinogenesis was firstly described by Roh et al. [166]. *MGMT* promoter hypermethylation was detected in 12 of 86 (14.0%) EOCs and strongly negatively correlated with *MGMT* expression. These data suggest that in sporadic OvC, *MGMT* is repressed mainly due to methylation of its promoter. Meta-analysis of 10 studies comprising 910 ovarian tissue samples by Qiao et al. concluded that the inactivation of *MGMT* might be associated with carcinogenesis in specific histological types of EOC [26]. Aberrant *MGMT* promoter methylation appears also in other human cancers such as cervical cancer [167], lung cancer [168], and glioblastoma [169].

As a DNA repair protein, MGMT seems to be also implicated in OvC chemoresistance. It was found to transcriptionally activate deubiquitinating enzyme 3 (DUB3) which stabilizes myeloid cell leukemia 1 (MCL1), an anti-apoptotic protein belonging to B-cell lymphoma 2 (BCL2) protein family [170]. This upregulation of *MCL1* prevents apoptosis and is essential for tumors to evade anti-cancer drugs and become resistant. To suppress the growth of *MGMT*-*DUB3*-*MCL1*-overexpressing cells may be useful a combined therapy with histone deacetylase inhibitors and O^6^-(4-bromothenyl)guanine (PaTrin-2).

Altered expression of some members of the AlkB human homolog family, the latter mentioned group of dealkylating enzymes, has been related to OvC at the level of post-transcriptional modification to mRNA. Methyl modifications to mRNA allow post-transcriptional control of gene expression by altering the mRNA interactions with other cell components [171]. Demethylation of the *N^1^* atom of adenine by AlkB homolog 3 (AlkBH3) was found to increase the half-life of colony-stimulating factor 1 (CSF1) mRNA without affecting the translation efficiency [172]. The expression of cytokine *CSF1* predicts poor prognosis in ovarian and breast tumors [173]. Also, another AlkB homolog 5 (AlkBH5) was found to enhance the stability of BCL-2 mRNA through demethylation of *N^6^* atom of adenine in EOC [174].

## 4. Interplay of DNA Repair Pathways

**Figure 8:**
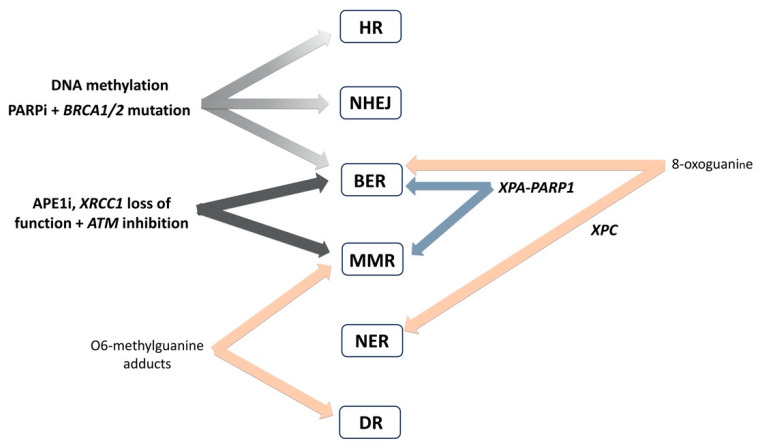
Interplay of DNA repair pathways. Simplified scheme of interactions between proteins from distinct DNA repair pathways (genes interacting in OvC in **bold**). Gene names: breast cancer 1 and 2 (*BRCA1* and *2*), X-ray repair cross-complementing 1 (*XRCC1*), Ataxia telangiectasia mutated (*ATM*), xeroderma pigmentosum complementation group A and C (*XPA* and *C*), poly(ADP-ribose) polymerase 1 (*PARP1*). Protein inhibitors: apurinic/apyrimidinic endonuclease 1 inhibitors (APE1i), poly(ADP-ribose) polymerase inhibitors (PARPi).

In our recent review, we have presented DNA repair as a complex biological process that ensures cellular integrity and genomic stability [3]. It has been known for long that DNA repair consists of several distinct pathways restoring different types of DNA damage [175]. In recent years, there is growing evidence of interactions among proteins involved in distinct DNA repair pathways. Regarding OvC, these interactions are illustrated in Figure 8.

As postulated by Nagel et al., no single pathway efficiently repairs all types of DNA lesions and some lesions serve as substrates for more than one pathway [176]. Another evidence of the interplay of different DNA repair pathways was found for O^6^-methylguanine adducts, which may be removed by both direct reversal repair or converted by MMR in DSBs [124]. Furthermore, Melis et al. indicated that XPC is involved in the initiation of several DNA damage-induced cellular responses and functions in the removal of DNA oxidation damage, redox homeostasis, and cell cycle control [177]. In our study on chromosomal aberrations in healthy individuals, we documented several DNA repair gene–gene combinations evinced either in enhanced or decreased frequencies of chromosomal aberrations [178]. In the frame of OvC, the attention is paid mainly to PARP1. Interestingly, polymerase PARP1 that detects SSBs within BER, has been found to regulate the NER system by its association with XPA [160]. The XPA-PARP1 non-covalent interaction reduces the XPA binding affinity to DNA, whereas XPA directly stimulates PARP1 enzymatic activity. On the other hand, PARP1 inhibition suppressed the recruitment of XPA to sites of laser-induced damage [179]. Likewise, PARPi decrease PARP1-XPA associations and reduce chromatin binding of XPA, suggesting the close relationship of both BER and NER pathways [160]. There is emerging evidence of extensive interactions among proteins involved in distinct DNA repair pathways and it needs to be reflected when evaluating the cancer etiology, prognostic and predictive factors based on DNA repair and DDR [180,181]. Although the concerted action of various DNA repair pathways in tumorigenesis is postulated, there is however scarce experimental evidence on this interplay.

The interaction of various DNA repair pathways found its application in cancer therapy [182]. Targeted therapy based on inhibiting DNA repair/DDR pathways enables tailoring the treatment of patients with tumors lacking functions in above pathways (e.g., inhibition of a complementary DDR pathway selectively kills cancer cells with a defect in a particular DNA repair pathway, i.e., concept of synthetic lethality; [183]). The concept of synthetic lethality has been utilized mostly in *BRCA1*/*2* mutated OvC patients, treated with PARPi [184]. The complexity of DNA repair/DDR, as nicely illustrated by Brown et al. 2017 [185], offers the use of other inhibitors as well. For instance, inhibitors of CHK1 and CHK2 appeared as promising therapeutics for OvC, both as monotherapy or in combination with PARPi [186,187]. The other example is based on the interaction of ATM inhibition in combination with APE1 inhibitors (APE1i) or XRCC1 loss of function [188,189]. Furthermore, nicotinamide adenine dinucleotide may affect DNA methyltransferase 1 through the regulation of BRCA1 in OvC [190]. A contemporary study provides evidence that oxidative DNA damage can cause dynamic changes in DNA methylation in the *BRCA1* gene due to the crosstalk between BER and de novo DNA methylation [191]. Interactions between DNA repair and DNA methylation may impact cellular regulatory mechanisms and epigenetic regulations in general and their understanding may contribute to the understanding of the carcinogenic process. The interaction of various DNA repair pathways and also DNA methylation present very promising applications in cancer therapy and OvC treatment in particular.

## 5. Therapeutic Perspectives–Targeting of DNA Repair System in Ovarian Cancer

As stated earlier, first-line treatment of OvC is based on surgery, followed by combination therapy of platinum derivatives and taxanes (usually carboplatin with paclitaxel) [17,18]. However, despite the initial remission of the disease, 70–85% of patients will experience relapse with a median survival of the recurrent OvC being 12–24 months [192]. Currently, new therapeutic approaches are directly aimed at molecular targets and pathways, e.g., anti-angiogenic agents such as bevacizumab or pazopanib, inhibitors of growth factor signaling, folate receptor inhibitors, inhibitors of AKT signaling, immunotherapeutic approaches and PARPi [19,20]. Targeting DNA repair has become a contemporary treatment option in OvC and it is aimed at DNA damage sensing, coordination of DNA repair, initiation of signaling pathways to promote cell cycle checkpoint activation, and triggering apoptosis [185].

PARPi have recently emerged as a promising class of new anti-cancer therapeutic agents. The employment of PARPi is a modern example of a synthetic lethality concept, based on alterations in DNA repair pathways. For instance, inhibition of PARP1 enzyme, a part of BER, results in persistent SSBs, the subsequent collapse of the replication fork, and the ultimate formation of DSBs. If this inhibition is applied in OvC tumors with defective HR, tumor cells utilize error-prone NHEJ, leading to the accumulation of DNA damage and cell death [144]. Since 2014, three PARPi have been approved by FDA and EMA for use in OvC–olaparib, rucaparib, and niraparib [144]. Another PARPi—veliparib and talazoparib—are showing promising clinical results and facing FDA and EMA approvals in the treatment of OvC shortly [193,194,195].

Among the other DNA repair system targets, cell cycle checkpoints as an essential part of DDR machinery are the most promising targets. They provide cell cycle arrest during which cells activate appropriate DNA repair mechanisms and efficiently repair damaged DNA. Since defects in DNA repair pathways are a prominent feature of OvC tumors, targeting DDR is nowadays one of the most extensively studied therapeutic approaches. However, the current lack of impressive clinical responses to DDR inhibitors, in general, would presumably make DDR inhibitors a part of cancer combination therapy (with either pharmacological treatment and/or radiotherapy), with only limited use as single agents [185,196].

Into common DNA damage caused by irradiation comprises base damage, crosslinks, SSBs and mostly DSBs. Therefore, targeting DDR may lead to potentiation of radiotherapy. There are several studies showing the potential of combination therapy based on irradiation and various DDR inhibitors (DNA-dependent protein kinase (DNA-PK), ATM/ATR, LIG4, PARP1, CHK1) but mainly in other types of cancer [197]. Radiotherapy is one of the least used therapeutic methods in OvC treatment at present. Although majority of the OvC is radiosensitive, the topographical position of ovaries in peritoneal cavity with other organs, which are rather radiosensitive, limits the applications of radiotherapy. High rates of both acute and chronic toxicity, especially gastrointestinal, lead to abandoning the treatment. With the discoveries of more potent chemotherapy drugs, radiotherapy is left for inoperable chemoresistant cases or for metastases [198].

Over 96% of HGSOC tumors are harboring gain-of-function or loss-of-function mutations in *TP53* (encoding p53 protein) leading to the disfunction of the G1/S phase checkpoint [199]. HGSOC tumors cells than heavily rely on G2/M checkpoint making it a possible target of anti-cancer therapy [186]. Inhibition of essential proteins involved in G2/M checkpoint may be exploited in anti-cancer therapy. Disabling of cell cycle arrest followed by mitosis may result in a mitotic catastrophe due to the lack of DNA repair and excessive DNA damage. Several DDR inhibitors have been studied in connection to OvC therapy, encompassing CHK1, ATR, ATM or Wee1-like protein kinase 1 (WEE1) inhibitors.

CHEK1 is a serine/threonine protein kinase which phosphorylates several downstream effectors including various proteins involved in cell cycle arrest, p53, DNA repair proteins, and proteins involved in cell death and transcription inhibition [200]. CHEK1 is an essential part of the G2/M checkpoint signaling pathway and it is overexpressed in almost all HGSOC [201], suggesting a need of cancer cells for G2/M checkpoint and arrest to essential DNA repair. Therefore, CHEK1 inhibitors (CHEK1i) are one of the most promising new therapeutic agents as suggested in Table 2. The CHEK1i V158411, PF-477736 and AZD7762 revealed efficiency in ovarian carcinoma cell lines [202,203]. In vitro and in vivo (on patient-derived xenograft mice models) studies revealed an extensive activity of the other potent CHEK1 (and CHEK2) inhibitor prexasertib in HGSOC, both as a monotherapy and in combination with PARPi olaparib, with anti-tumor activity even in olaparib-resistant models [204]. At present, prexasertib is being clinically tested as a therapeutic for OvC [187,201].

ATR is a central checkpoint kinase activated by DNA SSBs which may also result from the processing of DSBs and stalled replication fork. After activation, ATR phosphorylates a series of substrates promoting a wide array of cellular responses including activation of cell cycle checkpoints (via CHEK1 and WEE1), cell cycle arrest, DNA repair, and eventually apoptosis [205]. Several potent small molecules have been discovered to be used as ATR inhibitors (ATRi). In vitro study on ATRi (VE-821, VE-822, AZ20) shows that inhibition of ATR may resensitize PARPi-resistant cell lines to PARPi [206]. Recent in vitro study on PARPi-resistant OvC cell lines from Burgess et al. [207] confirms these results with ATR inhibitor VE-821, making treatment with ATRi a new promising approach to overcome PARPi-resistance in HR-deficient OvC. ATR inhibitor AZD6738 in combination with PARPi has revealed higher efficiency than PARPi alone [208,209].

**Table 2:** Current promising therapeutic approaches targeting DNA repair system in OvC.

DNA Repair Pathway	Gene Targets	In Vitro/In Vivo Efficiency	Pre-Clinical/Clinical Studies
Base Excision Repair	PARPi	Talazoparib and veliparib are in advanced clinical trials at the moment. Clinically available PARPi olaparib, rucaparib and niraparib are currently approved for the therapy of OvC on the basis of their *BRCA1*/*2* status (summarized in [210])	Olaparib-approved by FDA and EMA for use in OvC therapy [144] Rucaparib-approved by FDA and EMA for use in OvC therapy [144] Niraparib-approved by FDA and EMA for use in OvC therapy [144] Veliparib–advanced clinical trials in combination with carboplatin and paclitaxel. Veliparib induction therapy followed by veliparib maintenance therapy led to significantly longer PFS than carboplatin plus paclitaxel induction therapy alone [193,194] Talazoparib–ongoing advanced clinical trials [194,195]
Cell cycle checkpoints	CHEK1i	The CHEK1i V158411, PF-477736 and AZD7762 inhibited the proliferation of OvC cells [202] AZD7762 in combination with cisplatin suggested synergistic effects in ovarian clear cell carcinoma cell lines in vitro and suppressed growth of tumors in vivo [203] Prexasertib–effective in monotherapy in PARPi-resistant HGSOC cell lines and mouse xenografts [204] Combination of prexasertib mesylate monohydrate (LY2606368), a CHEK1 and CHEK2 inhibitor, and a PARPi, olaparib synergistically decreased cell viability in HGSOC cell lines (OVCAR3, OV90, PEO1 and PEO4) cell lines and induced greater DNA damage and apoptosis than the control and/or monotherapies [204,211]	Prexasertib–effective in clinical phase II study in recurrent HGSOC [201]
	ATRi	ATRi (VE-821, VE-822, AZ20) resensitized PARPi-resistant *BRCA1*-mutated human OvC cell line to PARPi [206] AZD6738 efficient in in *ATM*-deficient cells and in vivo in PDX mouse models with complete *ATM* loss [208] Combination PARPi with ATRi (AZD6738) and CHEK1i (MK8776) is more effective than PARPi alone in reducing tumor burden in *BRCA1*/*2* mutated HGSOC cells and PDX models [209]	Ongoing clinical PhaseII CAPRI Study of ATRi AZD6738 (ceralasertib) in combination with PARPi olaparib in HGSOC patients [212]
	ATMi	ATMi KU55933 enhanced the response to ionizing radiation in A2780 and OVCAR3 OvC cells [213]	
	WEE1i	Adavosertib (AZD 1775 alias MK1775)–efficient in vitro in SKOV-3 and ID8 OvC cell lines, efficient in vivo in ID8 ovarian tumors in monotherapy independent on *TP53* or *BRCA1* status [214]	AZD1775–active in phase I clinical study of monotherapy in OvC patients carrying *BRCA* mutations [215] AZD1775–combination therapy with AZD1775 enhanced carboplatin efficacy in *TP53*-mutated ovarian tumors in phase II clinical study [216]

Several clinical trials on the use of ATRi alone or in combination therapy (with PARPi or conventional chemotherapeutics) of OvC are in early initiation phases (for more see e.g., [212], Table 2) with results expecting in next few years.

By the presence of DSBs, ATM is activated as an essential part of DDR machinery. ATM phosphorylates hundreds of substrates to activate G1/S checkpoint, to induce intra-S and G2/M cell cycle arrest, DNA repair, chromatin remodeling, transcription, and apoptosis [205]. Mutations in *ATM* are known to cause Ataxia telangiectasia syndrome, a multisystem disorder characterized by progressive neurological impairment, immunodeficiency, hypersensitivity to X-rays, and predisposition to several cancers. Somatic mutations in *ATM* are present in several cancers including hematologic malignancies (e.g., are present in about 45% of mantle cell lymphoma cases), hepatocellular cancer, CRC, skin cancer, BC and others, however, only rarely mutated in OvC [217].

ATM inhibition has been shown to be synthetic lethal in vitro in combination with APE1i or functional loss of XRCC1 [188,189]. ATM inhibitors (ATMi) are known potent radio-sensitizers, studied currently on in vitro and in vivo models mainly for its potential use in brain-tumors cancer therapy [218,219,220]. However, in vitro results show that ATMi sensitize different gynecological cancer cell lines (e.g., A2780 and OVCAR3 ovarian cancer cells, Table 2) to ionizing radiation as well [213]. Additionally, a recent study from Riches et al. shows that AZD0156 (ATMi) enhances the effects of olaparib in lung, gastric and breast cancer cell lines and on triple negative breast cancer xenograft models [221], making it a potential tool in PARPi combination therapy in gynecological carcinomas. AZD0156 is currently being evaluated in phase I studies [221]. However, there is a limited amount of studies performed on OvC and further research is needed.

WEE1 mediates the activation of CDK1 and CDK2 kinases. Its increased gene expression has been observed in several cancers including OvC. High WEE1 protein levels are associated with poor survival in OvC patients with post-chemotherapy effusions, suggesting WEE1 inhibition may be a novel therapeutic approach in OvC [222]. Several in vitro studies on the role of a specific WEE1 inhibitor (WEE1i) adavosertib (AZD1775, MK1775) in combination therapies of several cancer models have been conducted [223,224]. Preclinical models showed a possible benefit of using WEE1i also with PARPi [225]. A recent study from Zhang et al. documented anti-tumor effects of adavosertib as a single agent in OvC therapy both in vitro and in vivo [214]. Still, the potential benefits of using WEE1i in OvC therapy have not been well established. Those data of recent studies suggest a high potential of various players in DNA repair/DDR pathways in OvC therapy.

## 6. Conclusions and Future Perspectives

Long term outcomes for OvC remains unsatisfactory (with five-year survival rates ranging from 30% to 50%) irrespectively of the advent of new treatment strategies. Based on the recent research activities it is completely clear that DNA repair machinery is involved in the risk of OvC development, the profile of the disease, and also in the prediction of therapeutic outcome. The functional status of DNA repair along with DDR determines cancer onset and impacts prognosis and efficacy of chemotherapy (often acting via DNA damage generation). The high-throughput genetic profile of DNA repair system genes allows us to identify and select crucial genetic variants important for prognosis and therapeutic response of OvC. However, the information on the prediction of therapeutic efficacy remains still fragmental, since many elements in the complex puzzle are missing. We have recorded that scarce studies address DNA repair in relation to the disease prognosis.

Our current review disclosed the following gaps in our understanding of the role of DNA repair/DDR in the onset, development, and management of OvC:

(i) We are facing the lack of systematic knowledge of DNA repair at various levels (i.e., genetic, epigenetic, protein, and functional) and their dynamic in the course of the disease. No available complex functional studies are characterizing any of the DNA repair pathways, as they do exist for other malignancies [226,227,228].
(ii) Although genetic alterations in HR repair pathway and their role in OvC are characterized decently, very little is known about the main pathway restoring DSBs, NHEJ. What is its importance in OvC onset, prognosis, and prediction? In the context of the previous point, further studies are needed on mechanisms (involvement of DSB repair?) underlying chromosomal instability in OvC (such as amplifications, deletions, translocations).
(iii) There is limited knowledge on the interaction of MMR (substantial in OvC etiology) with other DNA repair pathways. In this context, generally, more effort should be dedicated to the links between MMR (and other DNA repair pathways?) with immune response and with the microenvironment. These aspects may impact the patient’s prognosis, as they do in colon cancer.
(iv) In general, there is a poor understanding of interactions among individual DDR players.
(v) Contemporary studies illuminated interesting links between DNA damage, DNA repair, and DNA methylation/demethylation. This important aspect may exert future implications and consequences (epigenetic regulations).
(vi) Epigenetic regulation of DNA repair/DDR via non-coding RNAs should further be addressed in relation to the disease onset, prognosis, and therapy outcome.
(vii) There is a need to characterize OvC patients with a good and poor response with respect to the DNA repair system and its changes. Disclosure of critical determinants in DNA repair/DDR machinery could significantly contribute to the improvement of therapy success in OvC patients with multidrug-resistant tumors.

**Figure 9:**
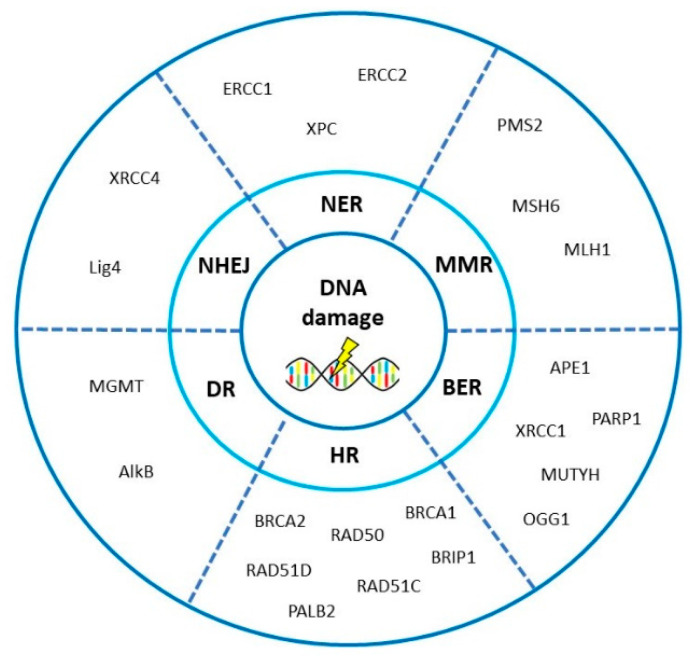
The most important genes involved in DNA repair pathways in ovarian cancer. Scheme of DNA damage and the most important genes playing role in ovarian carcinogenesis, prognosis and therapy response NER (nucleotide excision repair), BER (base excision repair), NHEJ (non-homologous end-joining repair), MMR (mismatch repair), HR (homologous recombination), DR (direct repair)). Protein names: PMS1 homolog 2 (PMS2), MutS homolog 6 (MSH6), MutL homolog 1 (MLH1), apurinic/apyrimidinic endonuclease 1 (APE1), poly(ADP-ribose) polymerase 1 (PARP1), X-ray repair cross-complementing 1 (XRCC1), MutY DNA glycosylase (MUTYH), 8-oxoguanine DNA glycosylase 1 (OGG1), breast cancer 1 and 2 (BRCA1 and 2), BRCA1-interacting protein C-terminal helicase (BRIP1), RAD50 homolog 1 (RAD50), RAD51 paralog C and paralog D (RAD51C and D), partner and localizer of BRCA2 (PALB2), alpha-ketoglutarate-dependent dioxygenase AlkB (AlkB), O^6^-methylguanine DNA methyltransferase (MGMT), DNA ligase 4 (LIG4), X-ray repair cross-complementing 4 (XRCC4), excision repair cross-complementation group 1 (ERCC1), xeroderma pigmentosum complementation protein C and D (XPC and D/ERCC2).

The imminent perspectives depend on addressing the above-listed points. The scientists/clinicians may reflect the axioms that alterations in DNA repair pathways (HR, MMR for instance) play a role in OvC, and targeting of DNA repair in a concept of synthetic lethality represents a beneficial therapeutic option. The most important genes of the DNA repair system in OvC (as illustrated in Figure 9 and described in Table 3) and their targeting in the frame of OvC will deserve further attention. The function of newly identified targets of DNA repair system in OvC therapy needs to be further defined. After that identification, targeted DNA repair gene manipulation may enable us to improve present clinically used regimens.

## Figures and Tables

**Table 1:** List of OvC-associated SNPs. Symbols: ↑ means higher, or better; ↓ means lower. Gene names: checkpoint kinase 2 (*CHEK2*), 8-oxoguanine DNA glycosylase 1 (*OGG1*), apurinic/apyrimidinic endonuclease 1 (*APE1*), X-ray repair cross-complementing 1 (*XRCC1*).

Gene	SNP	Functionality	Effect	Odds Ratio (OR), Hazard Ratio (HR), Confidence Interval (CI)	Population	Reference
*CHEK2*	rs17507066	Intron variant	↑ risk of serous EOC	OR: 0.86; 95% CI: 0.81–0.91	15,397 patients, 30,816 controls	[88]
	rs6005807	Intron variant	↑ risk of EOC	OR: 1.12, 95% CI: 1.07–1.18	15,397 patients, 30,816 controls	[88]
			↑ risk of serous EOC	OR: 1.17, 95% CI: 1.11–1.23	25,509 patients, 40,941 controls	[89]
*OGG1*	rs1052133	Missense variant, Ser326Cys	↑ risk	OR: 2.89; 95% CI: 2.47–3.38	720 patients, 720 controls	[90]
			↑ risk type II EOC	OR: 1.66; 95% CI: 1.26–2.17	420 patients, 840 controls	[91]
	rs2304277	Intron variant	↑ risk for BRCA1/2 carriers	HR: 1.12, 95% CI: 1.03–1.21	Stage I 1782 mutations carriers Stage II 23,463 mutations carriers	[92]
*APE1*	rs1130409	Missense variant, Asp148Glu	↓ risk	OR: 0.486; 95% CI: 0.344–0.688	124 patients, 141 controls	[93]
*XRCC1*	rs25487	Missense variant, Arg399Gln	↑ risk	OR: 2.54; 95% CI: 1.22–5.29	50 patients, 78 controls	[94]
			↑ risk of death	HR: 1.98; 95% CI: 1.09–3.93	195 patients	[95]
	rs1799782	Missense variant, Arg194Trp	↑ OS	HR: 0.61, 95% CI: 0.34–0.96	229 patients	[96]

**Table 3:** Overview of the most important DNA repair genes, their predisposition and prognostic impact and potential therapeutic use in targeted therapy for OvC. Symbols: ↑ means higher, or better; ↓ means lower, or worse. Protein names: breast cancer 1 and 2 (BRCA1 and 2), RAD51 paralog C and paralog D (RAD51C and D), RAD50 homolog 1 (RAD50), partner and localizer of BRCA2 (PALB2), BRCA1-interacting protein C-terminal helicase (BRIP1), PMS1 homolog 2 (PMS2), X-ray repair cross-complementing 4 (XRCC4), DNA ligase 4 (LIG4), MutS homolog 6 (MSH6), MutL homolog 1 (MLH1), PMS1 homolog 2 (PMS2), 8-oxoguanine DNA glycosylase 1 (OGG1), MutY DNA glycosylase (MUTYH), apurinic/apyrimidinic endonuclease 1 (APE1), X-ray repair cross-complementing 1 (XRCC1), poly(ADP-ribose) polymerase 1 (PARP1), xeroderma pigmentosum complementation protein C and D (XPC and D/ERCC2), excision repair cross-complementation group 1 (ERCC1), O^6^-methylguanine DNA methyltransferase (MGMT), alpha-ketoglutarate-dependent dioxygenase AlkB (ALKB).

DNA Repair Pathway	Gene	Predisposition Impact	Prognostic Impact	Therapeutic Potential (or Use)
Homologous recombination repair	*BRCA1*	Mutations associated with ↑ risk [45] and earlier onset [46]	↑ OS vs. non-carriers [50]	Better response to platinum-based chemotherapeutics [50,53], response to PARPi [55,229]
	*BRCA2*	Mutations associated with ↑ risk [45] and earlier onset [46]	↑ OS vs. non-carriers [50]	Better response to platinum-based chemotherapy [50,53], response to PARPi [55,229]
	*RAD51C*	Mutations associated with ↑ risk [59,60] and earlier onset [60]	N/A	Response to PARPi (in vivo and in vitro evidence) [64,65]
	*RAD51D*	Mutations associated with ↑ risk [9,61,62,63] and earlier onset [60]	N/A	Response to PARPi (in vivo and in vitro evidence) [65]
	*RAD50*	Mutated in about 0.12% of tumors [66]	Copy number deletion associated with ↑ OS and PFS [69]	In vitro knock-down associated with better response to PARPi [69]
	*PALB2*	Mutations associated with ↑ risk [73]	N/A	Response to PARPi (in vivo and in vitro evidence) [74,75]
	*BRIP1*	Mutations associated with ↑ risk [62,82,83,84]	N/A	Likely to predispose the response to PARPi and platinum [55]–needs further evaluation
Non-homologous end joining	*XRCC4*	N/A	↑ expression associated with ↓ OS [106]	N/A
	*LIG4*	Possible involvement of SNPs needs further evaluation	N/A	N/A
Mismatch repair	*MSH6*	N/A	N/A	Deficiency predisposes to platinum sensitivity in clear cell carcinoma [230]
	*MLH1*	Mutations associated with ↑ risk of Lynch syndrome-associated OvC [231]	↓ expression associated with ↑ OS and PFS [232]	N/A
	*PMS2*	Germline mutation associated with ↑ risk of Lynch syndrome-associated OvC [233]	N/A	N/A
Base excision repair	*OGG1*	SNPs associated with ↑ risk [90,91,132]	N/A	N/A
	*MUTYH*	Biallelic mutation associated with ↑ risk [135]	N/A	N/A
	*APE1*	SNP associated with ↑ risk [93]	↑ expression [139] and cytoplasmatic localization [140,141] have ↓ prognosis and OS	N/A
	*XRCC1*	SNP associated with ↑ risk [94]	SNPs [95,96,234,235] and ↑ expression [142] associated with ↓ prognosis	N/A
	*PARP1*	N/A	N/A	PARPi approved application for patients with germline BRCA1/2 mutations, with germline or somatic mutation BRCA1/2 with relapsed illness or with relapsed illness sensitive to platin-derivate chemotherapy regardless to BRCA status (FDA and EMA guidlines)
Nucleotide excision repair	*XPC*	N/A	SNPs associated with ↑ PFS [236]	N/A
	*XPD/ERCC2*	SNP associated with ↑ risk [237]	SNPs associated with prognosis [238]	SNP associated with severe neutropenia in patients treated by cisplatin-based chemotherapy [239]
	*ERCC1*	N/A	SNPs associated with ↑ OS [240]	SNP associated with ↑ risk of nephrotoxicity in patients treated by cisplatin-based chemotherapy [239]
Direct repair	*MGMT*	N/A	N/A	Likely to drive chemoresistance [170]
	*ALKB*	N/A	N/A	*ALKBH5* downregulation contributes to PARPi resistance in BRCA-deficient EOC [241]

## Abbreviations

APE1i	APE1 inhibitors
ATMi	ATM inhibitors
ATRi	ATR inhibitors
BER	base excision repair
CHEK1i	CHEK1 inhibitors
DDR	DNA damage response
DSB	double-strand break
EMA	European Medicine Agency
EOC	epithelial ovarian carcinoma
FDA	Food and Drug Administration U.S. agency
GWAS	genome wide association study
HGSOC	high-grade serous ovarian carcinoma
HR	homologous recombination repair
MMR	mismatch repair
MSI	microsatellite instability
NER	nucleotide excision repair
NHEJ	non-homologous end joining
OvC	ovarian cancer
OS	overall survival
PARPi	poly(ADP-ribose) polymerase inhibitors
PFS	progression-free survival
SNP	single-nucleotide polymorphism
SSB	single-strand break
WEE1i	WEE1 inhibitors
wt	wild-type
